# A pig model of acute right ventricular afterload increase by hypoxic pulmonary vasoconstriction

**DOI:** 10.1186/s13104-016-2333-7

**Published:** 2017-01-03

**Authors:** Kathrine Knai, Nils Kristian Skjaervold

**Affiliations:** 1 Department of Circulation and Medical Imaging, Faculty of Medicine, Norwegian University of Science and Technology, Trondheim, Norway; 2 Department of Circulation and Medical Imaging, Norwegian University of Science and Technology, Trondheim, Norway; 3 Department of Anaesthesia and Intensive Care Medicine, Trondheim University Hospital, Trondheim, Norway

**Keywords:** Acute right ventricular failure, Pulmonary hypertension, Hypoxic pulmonary vasoconstriction

## Abstract

**Background:**

The aim of this study was to construct a non-invasive model for acute right ventricular afterload increase by hypoxic pulmonary vasoconstriction. Intact animal models are vital to improving our understanding of the pathophysiology of acute right ventricular failure. Acute right ventricular failure is caused by increased afterload of the right ventricle by chronic or acute pulmonary hypertension combined with regionally or globally reduced right ventricular contractile capacity. Previous models are hampered by their invasiveness; this is unfortunate as the pulmonary circulation is a low-pressure system that needs to be studied in closed chest animals. Hypoxic pulmonary vasoconstriction is a mechanism that causes vasoconstriction in alveolar vessels in response to alveolar hypoxia. In this study we explored the use of hypoxic pulmonary vasoconstriction as a means to increase the pressure load on the right ventricle.

**Results:**

Pulmonary hypertension was induced by lowering the FiO_2_ to levels below the physiological range in eight anesthetized and mechanically ventilated pigs. The pigs were monitored with blood pressure measurements and blood gases. The mean pulmonary artery pressures (mPAP) of the animals increased from 18.3 (4.2) to 28.4 (4.6) mmHg and the pulmonary vascular resistance (PVR) from 254 (76) dyns/cm^5^ to 504 (191) dyns/cm^5^, with a lowering of FiO_2_ from 0.30 to 0.15 (0.024). The animals’ individual baseline mPAPs varied substantially as did their response to hypoxia. The reduced FiO_2_ level yielded an overall lowering in oxygen offer, but the global oxygen consumption was unaltered.

**Conclusions:**

We showed in this study that the mPAP and the PVR could be raised by approximately 100% in the study animals by lowering the FiO_2_ from 0.30 to 0.15 (0.024). We therefore present a novel method for minimally invasive (closed chest) right ventricular afterload manipulations intended for future studies of acute right ventricular failure. The method should in theory be reversible, although this was not studied in this work.

**Electronic supplementary material:**

The online version of this article (doi:10.1186/s13104-016-2333-7) contains supplementary material, which is available to authorized users.

## Background

Heart failure with subsequent circulatory shock is usually ascribed to the left ventricle and the systemic circulation. However, in recent years much attention has been paid to acute right ventricular failure (ARVF) and its contribution to circulatory shock, especially in intensive care medicine [1]. ARVF is most often caused by increased afterload of the right ventricle (RV) by chronic or acute pulmonary hypertension (PH) combined with regionally or globally reduced contractile capacity of the RV, although ARVF in the absence of PH is possible [2].

The pulmonary circulation is a low-pressure, low-resistance system. An increase in pulmonary resistance instantaneously raises the RV end-systolic pressure and volume [2–4]. This prolongs the isovolumic contraction, shortens the ejection phase and results in a pumping mechanism similar to that of the left ventricle. The result is both higher oxygen consumption and reduced oxygen supply, as coronary perfusion is diminished by elevated intramyocardial pressure. A normal RV can adapt to both chronic and, to a certain degree, acute afterload increases by hypertrophy. However, when the RV fails to compensate for the increased afterload, devastating progressive ARVF and total circulatory breakdown eventually develops.

There is certainly a need for more research on the mechanisms leading to ARVF as well as how to treat the condition [2]. With its complex nature, and because it is caused by relatively small pressure changes, it is important to use good intact animal models to study the phenomenon. Several previous large animal models have used open chest approaches [5–7]. We believe it is vital to keep the chest closed and leave the pericardium intact as these surgical manipulations alter the pressure-flow relations of the RV and the pulmonary circulation, and hence, disturb the ARVF model.

We therefore aimed to construct a new and feasible large animal model where the afterload of the RV could be changed—preferably reversibly—with minimal invasiveness and closed chest. In such a model the hemodynamic effect of increased afterload on RV can be studied in detail, and it would be possible to include this method in different models of RV failure.

In the pulmonary vasculature, vasoconstriction occurs in response to hypoxia, termed hypoxic pulmonary vasoconstriction (HPV). The mechanism acts in response to low alveolar pO_2_ and is a vital physiological function to direct blood to the ventilated alveoli, and thereby substantially reduce pulmonary shunting. With a decrease in alveolar pO_2_, HPV onsets within seconds [8], increases rapidly and gradually plateaus. The constriction is mainly localized in small resistance pulmonary arteries (PAs, <200 µm) [9] and is reversible with restoration of alveolar normoxia. Global hypoxia will induce HPV in all lung segments, resulting in a rise in pulmonary vascular resistance (PVR), with estimates ranging from 50 to 300% [10]. HPV has been used as a means to increase both acute and chronic PVR in animal experiments [11], but there is no systematic description in the literature on how to use HPV to induce acute RV afterload increase in pigs.

In this study we explored the use of HPV as a means to increase the pressure load on the RV aiming to determine the fraction of inspired oxygen (FiO_2_) level yielding the desired effect without leading to serious dysoxia. We used the standard definition of PH as a mean pulmonary artery pressure (mPAP) of ≥25 mmHg at rest as the target level [12].

## Methods

### Animals, handling, anaesthesia, surgery and euthanasia

Eight outbred pigs (*25% Duroc, 25% Yorkshire, 50% Norwegian Landrace*, 29–37 kg) were included in the study after approval from the Norwegian State Commission for Animal Experimentation. All the animals received human care in accordance with the European Convention for the Protection of Vertebrate Animals used for Experimental and Other Scientific Purposes.

The animals were premedicated with intramuscular diazepam 10 mg and azaperone 400 mg. Anaesthesia was induced through an intravenous access on the external ear with atropine 1.0 mg, fentanyl 8.0 μg/kg, thiopenthal sodium 4.0 mg/kg and ketamine hydrochloride 8.0 mg/kg. Before intubation, 5 ml of 40 mg/ml lidocaine was applied to the larynx. The animals were ventilated in PRVC mode on a Servo-i respirator (Maquet, Getinge Group, Gothenburg, Sweden) with initial values of FiO_2_ at 0.30, a tidal volume of 10 ml/kg, PEEP at 6 cmH_2_O and minute ventilation adjusted to maintain PaCO_2_ at 4.5–5.5 kPa. Anaesthesia was maintained by an infusion of fentanyl 20 μg/(kg h) and midazolam 0.40 mg/(kg h). Based on clinical response this was supplemented with boluses of fentanyl 50 μg/ml as needed. Intravascular volume was maintained by a bolus of acetated Ringer’s solution 10 ml/kg, followed by a continuous infusion of 10 ml/(kg h) throughout the experiment. 5000 IU heparin was administered i.v. to prevent clot formation.

Before cannulation we surgically prepared the left carotid artery and the right and left internal jugular vein. Two mono lumen catheters were inserted into the left carotid artery and the left internal jugular vein for invasive blood pressure monitoring, arterial blood gases and intravenous injections. A flow-directed pulmonary artery catheter (PAC; Swan Ganz CCOmbo 7.5 Fr, Edwards Lifescience, USA) was inserted in the right internal jugular vein and advanced into the pulmonary artery using classical pressure observations and fluoroscopic guidance and validation. The PAC provided central venous pressure (CVP), pulmonary artery pressure (PAP) and was used to sample mixed venous blood gases. The PAC was connected to a Vigilance II monitor for continuous cardiac output (CO) measurements (Edwards Lifescience, USA). A bladder catheter was inserted by a mini-laparotomy.

At the end of the experiment, the animals were euthanized with pentobarbital 100 mg/kg.

### Study protocol and respirator manipulations

To induce HPV, the FiO_2_ had to be reduced to levels below the physiological range (the oxygen content in air at sea level is 20.9%, and equals an FiO_2_ of 0.21). In order to reduce the FiO_2_ to subnormal values, nitrogen was plugged into the air inlet of the respirator, and the animals were then ventilated with an oxygen and nitrogen mixture. The actual FiO_2_ was monitored via a side stream multi-gas analyser.

After end of surgery the animals stabilized for 30 min before baseline measurements were recorded. PH was then induced by slowly reducing FiO_2_ on the respirator while monitoring the response in mPAP. When mPAP levels above 25 mmHg were reached, the animal rested for 30 new minutes before new measurements were recorded.

### Data material, measurements and statistics

Vital variables [mean pulmonary artery pressure (mPAP), pulmonary wedged pressure (PWP), central venous pressure (CVP), heart rate (HR), cardiac output (CO)] and arterial and mixed venous blood gases were measured at baseline and after establishing PH. Blood gases were processed by a Radiometer ABL 720 blood gas analyser (Radiometer, Brønshøj, Denmark).

Equations for calculating PVR, DO_2_ and VO_2_:

$$Pulmonary\,\,vascular\,\,resistance \,\,(PVR) = \frac{{80\left( {mPAP - PWP} \right)}}{CO}{\text{ Unit: dyn}} \,{\text{s}}/ {\text{cm}}^{5}$$

$$\begin{aligned} Arterial\,\,oxygen\,\,content\,\, ( {CaO_{2} }) & = \left( {Hgb \times 1.34} \right)SaO_{2}\\&\quad + ( {PaO_{2} \times 0.0031}) \\ &\quad\quad{\text{ Unit: ml O}}_{ 2} / {\text{dl blood}} \end{aligned}$$

$$Mixed \,\, venous\,\, oxygen \,\,content\,\, ( {CvO_{2} }) = \left( {Hgb \times 1.34} \right)SvO_{2} + \left( {PvO_{2} \times 0.0031} \right)$$

$$Oxygen\,\,delivery \,\, ( {DO_{2} } ) = CaO_{2} \times CO \times 10{\text{ Unit: ml O}}_{ 2} / {\text{min}}$$

$$Oxygen\,\, consumption \,\, ( {VO_{2} }) = C\left( {a - v} \right)O_{2} \times CO \times 10$$

Only simple descriptive medical statistics and paired sample t tests were used in this study. All datas were analysed in Excel (MS Excel for Mac 2011, Microsoft Corporation, USA) and R (version 3.1.1, The R Foundation for Statistical Computing, Vienna, Austria).

## Results

**Fig. 1:**
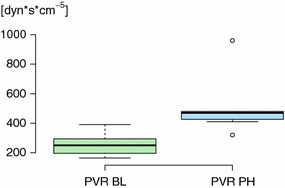
Changes in PVR from baseline to pulmonary hypertension. The overall increase in PVR from baseline to pulmonary hypertension is approximately 100%, with a p-value of 0.001. *PVR BL* pulmonary vascular resistance at baseline, *PVR PH* pulmonary vascular resistance at pulmonary hypertension

The individual animals had substantially different baseline mPAP values despite similar age, weight and handling, 18.3 (4.2) mmHg [mean (sd)] (range 14–27 mmHg). The FiO_2_ level needed to increase mPAP above 25 mmHg also varied considerably, 0.15 (0.026) [mean (sd)], range 0.13–0.21. (In the one animal with a baseline mPAP above 25 mmHg, the FiO_2_ was lowered to 0.15.) This yielded a highly significant overall increase in mPAP from 18.3 (4.2) to 28.4 (4.6) mmHg (p ≪ 0.0001) (Table 1). Since CO did not change, the change in PVR was also consistent in all animals: from 254(76) dyns/cm^5^ to 504(191) dyns/cm^5^, yielding a *p*-value of 0.001 (Fig. 1).

**Table 1:** Changes in mPAP, PWP and PVR from baseline to pulmonary hypertension

Pig	Baseline (BL)					Pulmonary hypertension (PH)				
	FiO_2_	mPAP (mmHg)	PWP (mmHg)	CO (l/min)	PVR (dyn s/cm^5^)	FiO_2_	mPAP (mmHg)	PWP (mmHg)	CO (l/min)	PVR (dyn s/cm^5^)
1	0.30	22	11	3.5	251.4	0.15	34	11	3.9	471.8
2	0.30	17	7	3.2	250.0	0.21	31	7	4.0	480.0
3	0.30	17	7	2.4	333.3	0.13	25	7	3.0	480.0
4	0.30	14	7	2.2	254.5	0.13	25	5	3.9	410.3
5	0.30	17	7	4.5	177.8	0.14	26	8	4.5	320.0
6	0.30	15	8	3.4	164.7	0.14	25	8	2.9	469.0
7	0.30	27	6	4.3	390.7	0.15	36	6	2.5	960.0
8	0.30	17	9	3.0	213.3	0.15	25	9	2.9	441.4

Both mPAP and PVR increase with reduction of FiO_2_ and globally induced hypoxic pulmonary vasoconstriction

*FiO*_*2*_ fraction of inspired oxygen, *mPAP* mean pulmonary arterial pressure, *PWP* pulmonary wedged pressure, *CO* cardiac output, *PVR* pulmonary vascular resistance

**Fig. 2:**
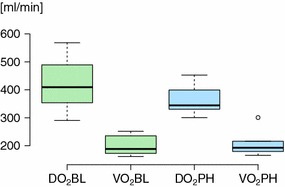
Changes in oxygen delivery and consumption from baseline to pulmonary hypertension. Neither DO_2_ nor VO_2_ changed with the reduction of FiO_2_ indicating minor disturbance to the model. *DO*_*2*_ *BL* oxygen delivery at baseline, *VO*_*2*_ *BL* oxygen consumption at baseline, *DO*_*2*_ *PH* oxygen delivery at pulmonary hypertension, *VO*_*2*_ *PH* oxygen consumption at pulmonary hypertension

**Fig. 3:**
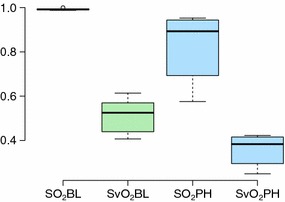
SO_2_ and SvO_2_ before and after decreased FiO_2_. There was some reduction with a large spread in SO_2_, and a large and significant change in SvO_2_ from baseline to pulmonary hypertension

Blood gases taken at baseline and after reduction of FiO_2_ displayed a slight reduction in oxygen delivery (DO_2_) from 409 (92) ml/min to 354 (52) ml/min. This was not a statistically significant reduction with a p value of 0.14. Nor did it affect the oxygen consumption (VO_2_), which remained unchanged, from 195 (35) ml/min to 200 (41) ml/min (Fig. 2). This was caused by increased oxygen extraction after induction of PH, with a highly significant reduction in mixed venous oxygen saturation (SvO_2_) from 0.51 (0.076) to 0.36 (0.070), yielding a p - value of ≪0.0001 (Fig. 3). Additionally, there were no changes in lactate, pH or base excess from baseline to after FiO_2_ reduction. For details, see Additional file 1.

## Discussion

Reduction of FiO_2_ from 0.3 to 0.15 (0.024) is an efficient method for increasing the afterload of the RV, yielding an overall doubling of the PVR. The effect took place in all animals and should therefore be considered a reliable general physiological feature. As such, this method can be used in future studies of RV adaption to increased afterload; the combination with some method of regional or global RV contractility reduction should in theory induce ARVF (Additional file 1: Table S1).

There was considerable variation in individual animals’ baseline mPAPs and the FiO_2_ reduction needed for a sufficient mPAP increase. One could therefore ask whether this kind of model should aim at increasing the mPAP (or PVR) above a clinical definition of PH, as was done in this study, or whether one should aim for a relative change in afterload in each respective animal, say, a 100% increase in mPAP (or PVR) from baseline. Either way, the FiO_2_ reduction has to be adapted to the individual animal.

Some animals experienced a relatively large reduction of arterial oxygen saturation (SaO_2_) as a consequence of the FiO_2_ reduction, leading to decreased DO_2_ overall. This did not lead to a decreased VO_2_ because of an increased oxygen extraction. It should be noted, however, that this occurred in the stable situation 30 min after establishing the lower FiO_2_ value (Fig. 2). We noticed during the initial phase of FiO_2_ reduction a transient change in skin colour and a decline in plethysmographic measured SaO_2_. After some minutes with their new oxygen supply, the animals regained their previous colour and SaO_2_ level. This immediate physiological instability after serious system perturbations followed by stabilization after a few minutes is an interesting physiological feature that we have also experienced in other animal models.

Another word of caution is that in some of the pilot animals prior to this study, we lowered the FiO_2_ too rapidly to levels that were too low, yielding an extremely low SvO_2_. This is a very strong stimulus to the sympathetic nervous system, leading to tachycardia and a high CO to compensate for the hypoxia. The pulmonary circulation is constructed to be able to receive large volumes of blood without increasing the pulmonary arterial pressure. This is mainly caused by an increase of the total cross-sectional area of the pulmonary vasculature, resulting from passive distension and recruitment of collapsed vessels [13]. In this situation the animals ended up with an increased mPAP from baseline, but with the same or even a reduced PVR. It is therefore important to be very careful when using our method, because it approaches the limit of the animal’s ability to compensate.

In theory, this method of increasing mPAP and PVR by reducing FiO_2_ should be fully reversible. Unfortunately, we did not study whether the mPAP returned to its baseline value after re-establishing normal FiO_2_, or the time frame needed for this to occur. In the literature, HPV is described as biphasic with one rapid and one late phase. The time aspect is inconclusively described with variations in onset and duration for both phases [10, 13–15]. The long phase is said to last from 45 min to several hours, but there are no conclusive descriptions of how long the hypoxia must be maintained for this phase to kick in, nor how fast the vascular tone is returned to normal with restoration of normoxia. This emphasizes the need for further investigation of the method’s reversibility.

This study was not designed to investigate RV changes as a consequence of increased afterload. In accordance with the theory as described in the background, increased afterload itself should not be enough to cause failure in a healthy RV. This was also our impression during the study, as CVP remained unchanged and CO didn’t show any systematically decrease. However, a full assessment of RV’s adaption to changing loading conditions requires assessment with conductance catheters or similar in order to extract pressure–volume loops [6, 11].

*Resistance* as defined in practical clinical medicine and in this manuscript as a measure of afterload is an application of Ohm’s law where resistance = Δ pressure/flow. In reality, this is only valid for a system of linear flow. In the pulmonary (and systemic) circulation, flow is oscillatory, in which *impedance* is a better calculation of the overall afterload. The continuous flow and pressure signals are assessed by Fourier analyzes and then the pressure/flow relations are compared for the respective harmonics [16]. Thus, in order to examine impedance, high-resolution continuous pressure and flow signals are mandatory, which we did not have in this study.

## Conclusion

We showed in this study that the mPAP and the PVR could be raised by approximately 100% in the study animals by lowering the FiO_2_ from 0.30 to 0.15 (0.024). The responses were individual, as were the baseline mPAPs. This FiO_2_ reduction led to reduced SaO_2_ and hence DO_2_ in some animals, but the VO_2_ was not reduced after a stabilization period. We note that in the initial phase, the animals seemed to be unstable, with clinical signs of dysoxia, but they regained normal oxygenation after some minutes. Furthermore, this method should be used with some caution, as reducing FiO_2_ too rapidly might lead to sympathetic nervous activation with tachycardia and high CO, resulting in a secondary normalization of PVR despite high mPAP. The method should in principle be reversible, although we did not study this in this work. We therefore present a novel method for minimal-invasive (closed chest) RV afterload manipulations intended for future studies of ARVF.

## Additional file

**Additional file 1: Table S1.** Arterial and mixed venous blood gases at baseline and pulmonary hypertension.

## Abbreviations

ARVF: acute right ventricular failure
RV: right ventricle
PH: pulmonary hypertension
HPV: hypoxic pulmonary vasoconstriction
FiO_2_: fraction of inspired oxygen
PAC: pulmonary artery catheter
PAP: pulmonary artery pressure
PWP: pulmonary wedged pressure
CVP: central venous pressure
HR: heart rate
CO: cardiac output
PVR: pulmonary vascular resistance
DO_2_: oxygen delivery
VO_2_: oxygen consumption
