# Supplementary material for: A pig model of acute right ventricular afterload increase by hypoxic pulmonary vasoconstriction
Source: BMC Res Notes. 2017 Jan 3;10:2. doi: 10.1186/s13104-016-2333-7 (PMC5217654; doi:10.1186/s13104-016-2333-7)
Supplement: Supplementary file 1 — Additional file 1: Table S1. Arterial and mixed venous blood gases at baseline and pulmonary hypertension. [file 13104_2016_2333_MOESM1_ESM.pdf]

Arterial and mixed venous blood gases at baseline and pulmonary hypertension:

	Baseline									Pulmonary hypertension								
	**CO [L/min]**	**pH**	**Hb [g/dL]**	**PaO_2 [kPa]**	**PvO_2 [kPa]**	**SaO_2**	**SvO_2**	**DO_2 [$dynscm^{-5}$]**	**VO_2 [$dynscm^{-5}$]**	**CO**	**pH**	**Hb [g/dL]**	**PaO_2 [kPa]**	**PvO_2 [kPa]**	**SaO_2**	**SvO_2**	**DO_2 [$dynscm^{-5}$]**	**DO_2 [$dynscm^{-5}$]**
**Pig 1**	3,5	7,5	9,5	17,8	4,05	99,4 %	43,3 %	444,8	251,4	3,9	7,5	10,3	5,37	3,10	67,2 %	27,3 %	362,4	215,0
**Pig 2**	3,2	7,45	9,7	19,10	4,30	99,0 %	44,6 %	413,7	227,7	4,0	7,5	8,9	9,57	3,36	94,7 %	31,8 %	452,9	300,8
**Pig 3**	2,4	7,57	10,2	22,80	4,39	99,1 %	50,4 %	326,8	161,1	3,0	7,57	9,9	7,10	3,90	86,4 %	42,1 %	344,5	176,6
**Pig 4**	2,2	7,48	9,8	19,9	3,94	100,0 %	40,7 %	290,3	172,4	3,9	7,43	11,4	5,07	3,27	57,6 %	24,9 %	343,8	195,0
**Pig 5**	4,5	7,41	9,5	19,4	5,51	98,8 %	56,7 %	568,7	243,1	4,5	7,46	10,1	6,05	3,87	71,5 %	36,0 %	436,3	216,5
**Pig 6**	3,4	7,46	8,9	21,6	4,97	99,4 %	57,3 %	405,3	172,5	2,9	7,54	9,1	7,79	3,64	92,2 %	40,7 %	326,7	182,5
**Pig 7**	4,3	7,49	9,3	21,20	5,52	99,1 %	61,4 %	533,9	204,1	2,5	7,58	9,5	8,78	4,00	94,1 %	42,2 %	300,2	165,5
**Pig 8**	3,0	7,53	9,5	22,10	4,87	99,2 %	54,6 %	380,9	171,9	2,9	7,58	9,0	9,59	3,86	95,3 %	41,0 %	334,2	190,4

CO, cardiac output; **Hb**, hemoglobine; **PaO_2**, arterial oxygen partial pressure; **PvO_2**, mixed venous oxygen partial pressure; **SaO_2**, arterial oxygen saturation; **SvO_2**, mixed venous oxygen saturation; **DO_2**, oxygen delivery; **VO_2**, oxygen consumption.
